# Supplementary material for: Sensing leg movement enhances wearable monitoring of energy expenditure
Source: Nat Commun. 2021 Jul 13;12:4312. doi: 10.1038/s41467-021-24173-x (PMC8277831; doi:10.1038/s41467-021-24173-x)
Supplement: Supplementary file 1 — Supplementary Information [file 41467_2021_24173_MOESM1_ESM.pdf]

Supplementary Materials

Supplementary Fig.1. Experimental conditions and sensors collected to select the best estimation method.
Supplementary Fig.2. Visual illustration of the Data-Driven Model.
Supplementary Fig.3. Comparison of methods estimating steady-state energy expenditure.
Supplementary Fig.4. Extending offline estimation to time-varying activity.
Supplementary Fig.5. The Data-Driven Model error as a function of training data.
Supplementary Fig.6. Estimating steady-state energy expenditure with limited training data.
Supplementary Fig.7. Steady-state errors for each activity and method.
Supplementary Table 1. The Data-Driven Model results for permutations of input sensor classes.
Supplementary Table 2. The Data-Driven Model results for permutations of inertial measurement units placed at different locations on one leg.
Supplementary Table 3. Computation time for the Wearable System and the Musculoskeletal Model.
Supplementary Table 4. System usability survey results.
Supplementary Table 5. System comfort survey results.

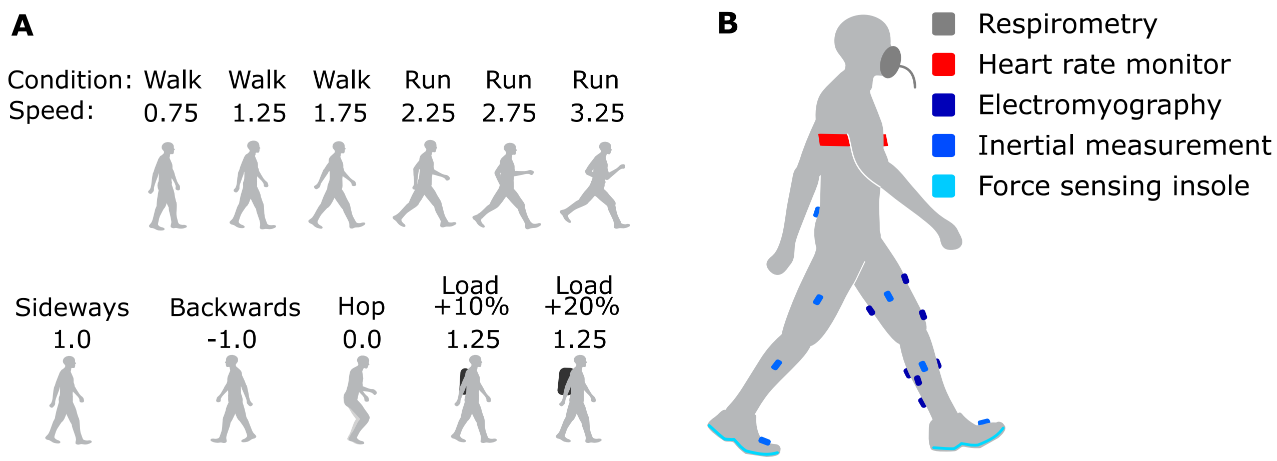

Supplementary Fig.1. Experimental conditions and sensors collected to select the best estimation method. (**A**) Eleven conditions and quiet standing were collected including a variety of walking, running, and hopping motions. (**B**) A variety of wearable sensor data and tethered Respirometry were collected.

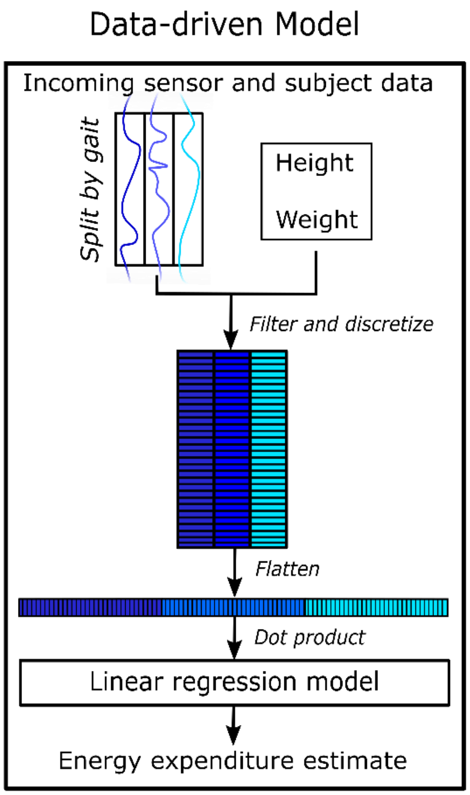

Supplementary Fig.2. Visual illustration of the Data-Driven Model. The Data-Driven Model processes input data by segmenting stride, filtering, discretizing to a fixed input size, and flattening the processed inputs to a vector. An estimate of energy expenditure is computed by passing the processed inputs to a trained linear regression model.

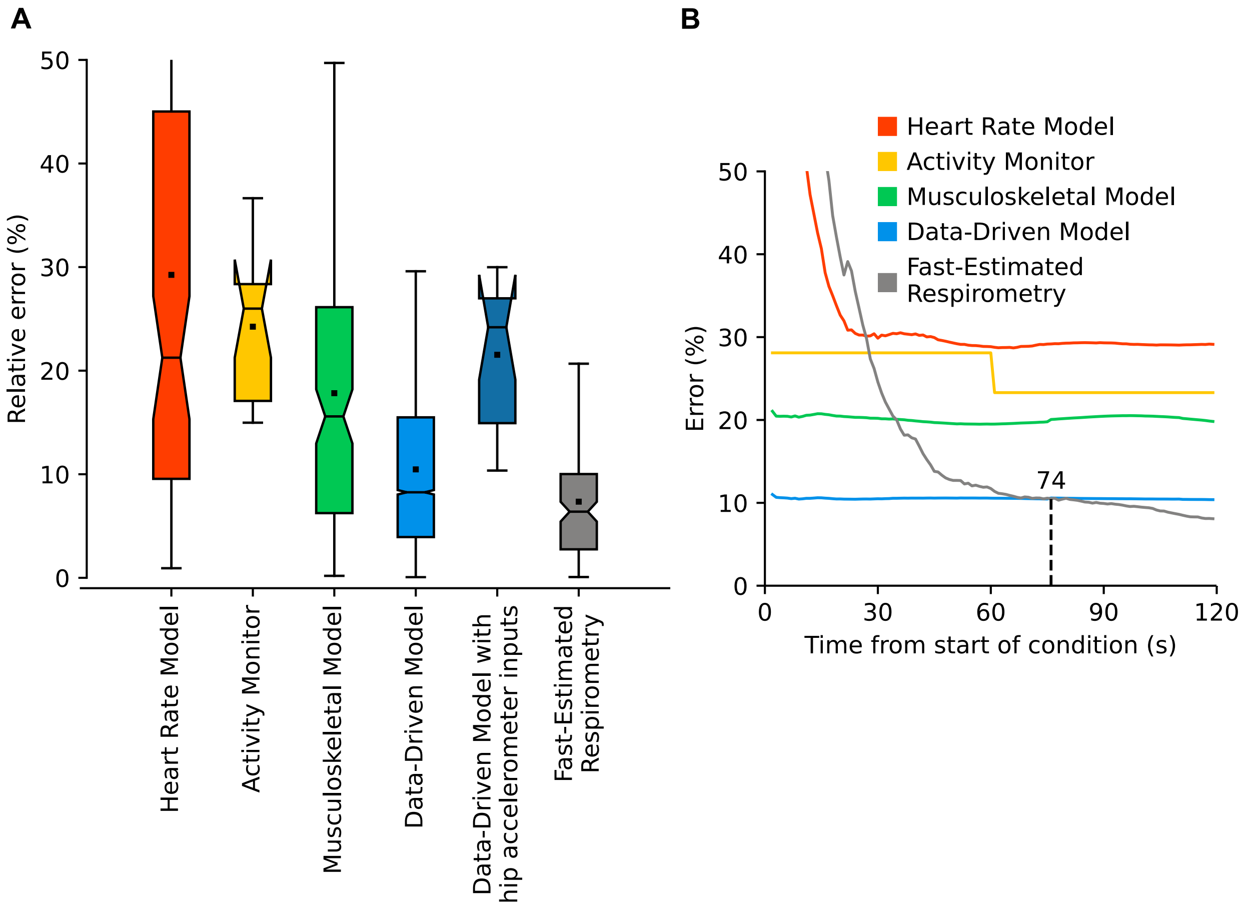

Supplementary Fig.3. Comparison of methods estimating steady-state energy expenditure. (**A**) The Data-Driven Model was selected as the best method because it had the lowest error of the wearable methods. Fast-Estimated Respirometry relies on the lab-based respirometry equipment which provides ground truth energy expenditure measurements. The Data-Driven Model relied on all wearable sensor inputs. Accuracy was evaluated using a cross-validation approach that held out one subject (n = 13) and one condition from the training data, fit the method with the training data if appropriate, and estimated energy expenditure for the withheld subject and condition. We did this for all permutations of subjects and conditions and averaged the error. The boxes extend from the lower to upper quartile values of the data, with a line at the median and a dot at the mean. The whiskers extend to the last data point within 2.5 times the interquartile range. Fliers were not plotted due to the wide range in errors. (**B**) Energy expenditure errors from the start of steady-state conditions were compared to understand how quickly each method converged to a steady-state estimate. The Data-Driven Model converged the fastest and had the lowest error initially. Fast-Estimated Respirometry had the lowest error after 74 seconds.

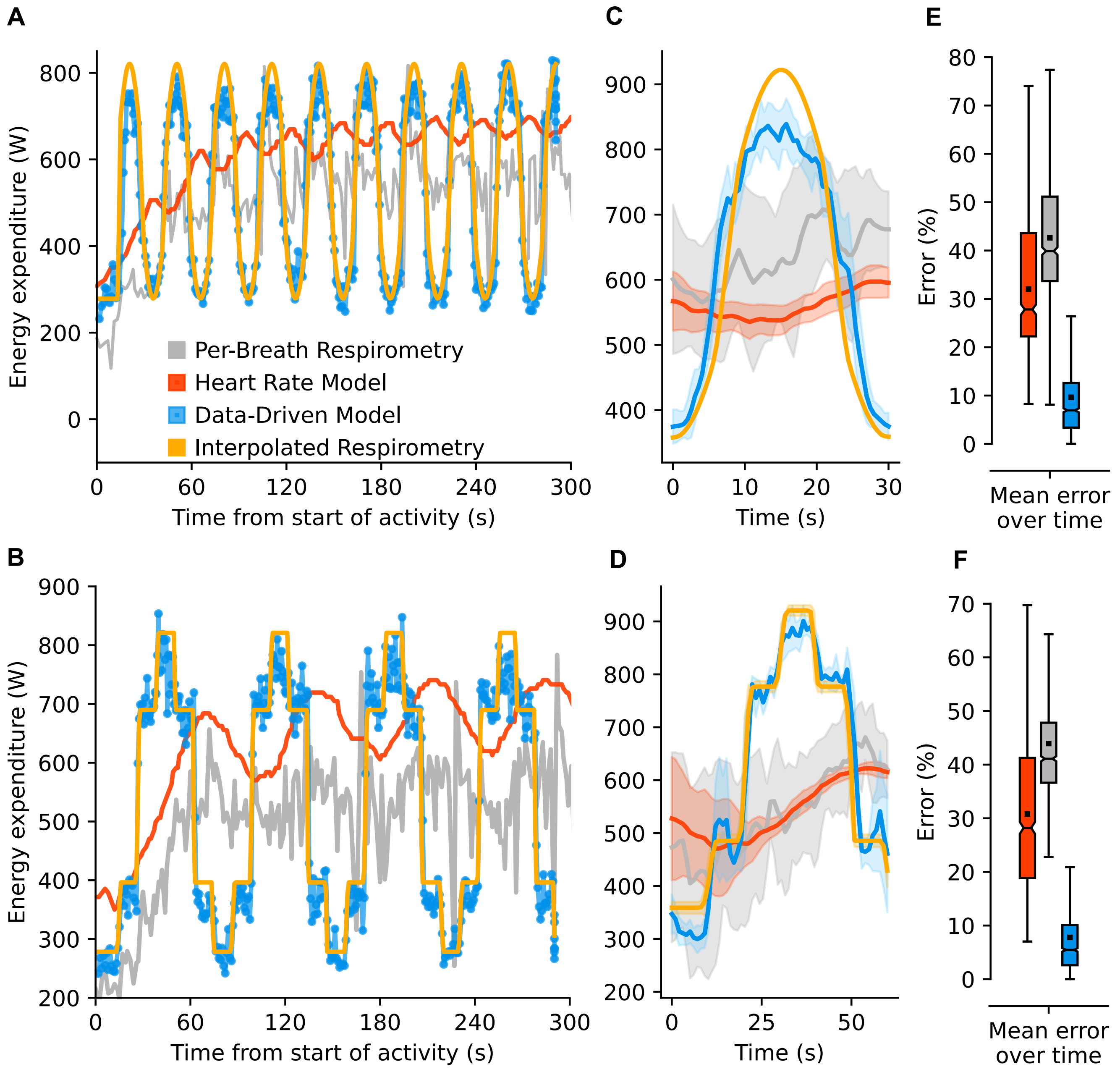

Supplementary Fig.4. Extending offline estimation to time-varying activity. The we compared methods of estimating energy expenditure with an experiment (n = 4) where subjects completed time-varying conditions following periodic changes in treadmill speed which were (**A**) varied sinusoidally or (**B**) with discrete steps between walking and running at 1.25 and 2.75 m/s. The Activity Monitor and Musculoskeletal model were not evaluated during time-varying conditions because of slow estimation rate and significant computation time (Supplementary Table 1), respectively. Data from a representative subject illustrates the estimates and ground truth Interpolated Respirometry. (**C**, **D**) The wearable data-driven estimates and Interpolated Respirometry were averaged over the cyclic periods to find the mean and standard deviation across subjects tested with this protocol. The error band represents one standard deviation. (**E**, **F**) The absolute error was computed at each time step and then averaged. The boxes extend from the lower to upper quartile values of the data, with a line at the median and a dot at the mean. The whiskers extend to the last data point within 2.5 times the interquartile range. Fliers were not plotted due to the wide range in errors.

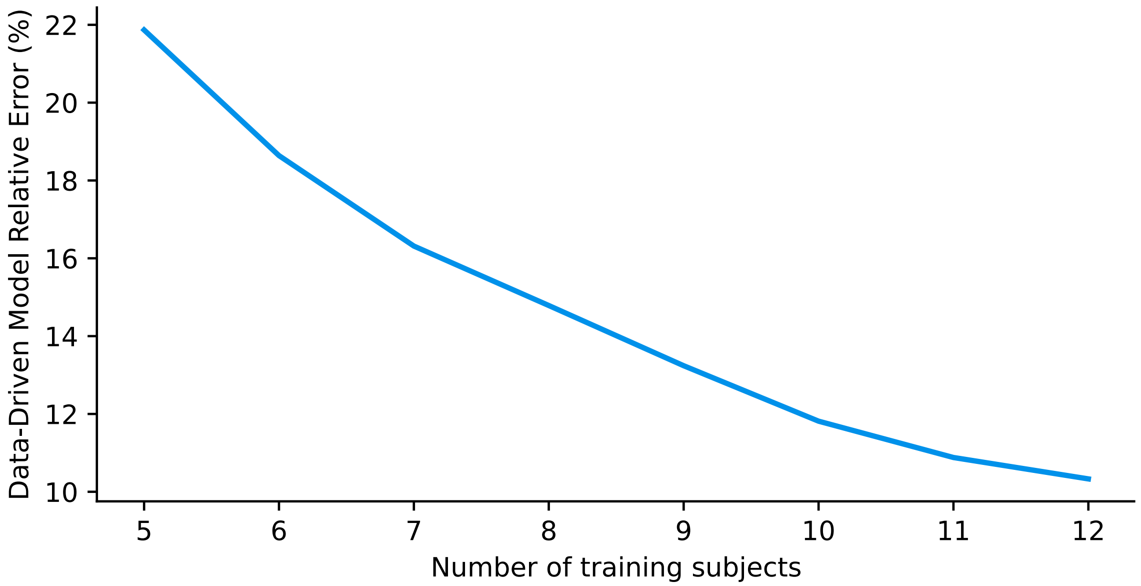

Supplementary Fig.5. The Data-Driven Model error as a function of training data. The error decreased with additional subjects included in the training data. The reduction in error with each model decreases, suggesting a limit to the error.

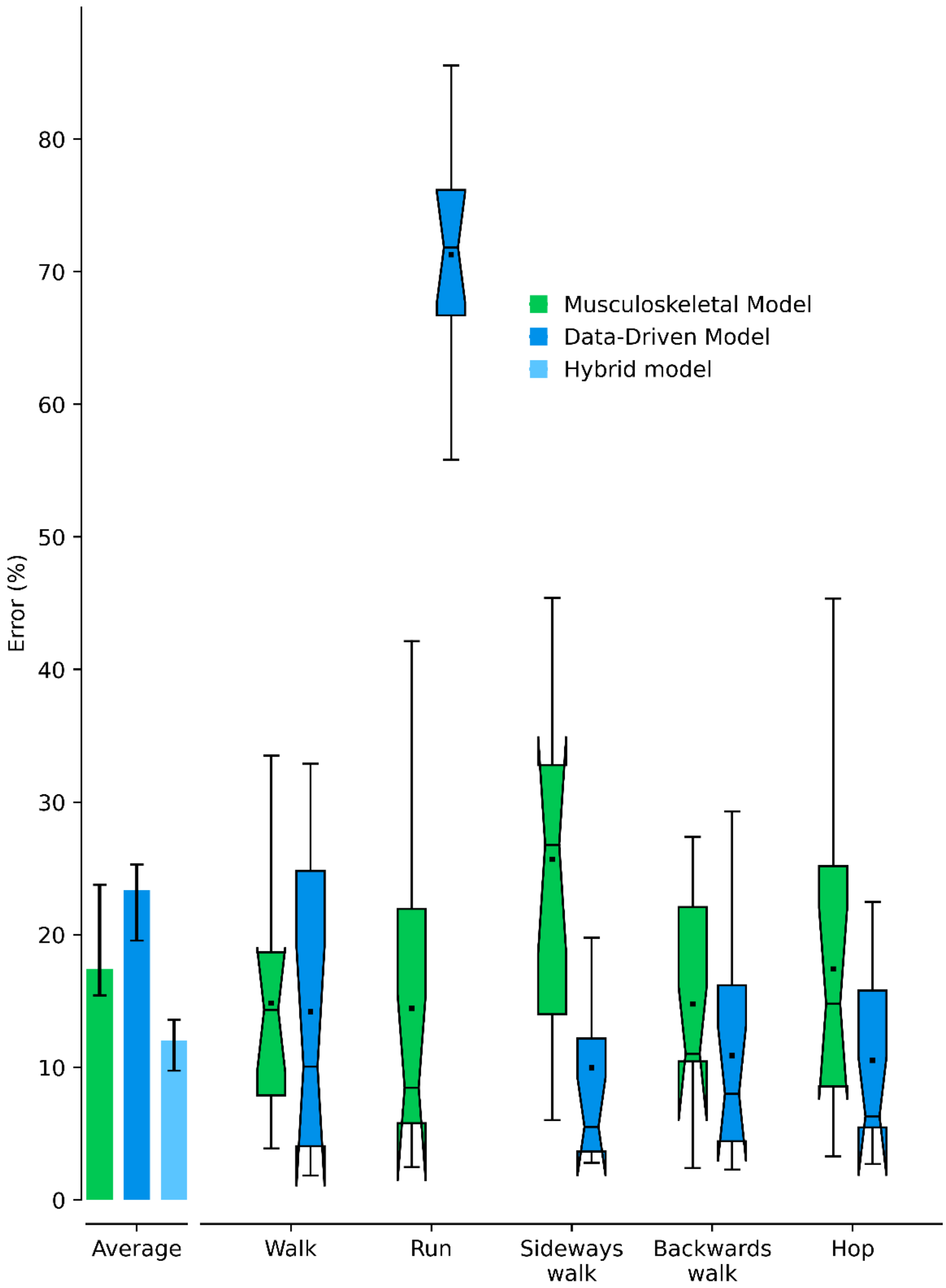

Supplementary Fig.6. Estimating steady-state energy expenditure with limited training data. The minimal dataset consisted of one condition per activity: walking at 1.25 m/s, running at 2.75 m/s, sideways walking at 1 m/s, backwards walking at 1 m/s, and self-selected hopping in place. The minimal dataset tested the methods on a new subject (*n* = 13) and new conditions using cross-validation. The Data-Driven Model had large error during running, resulting in worse overall error than the Musculoskeletal Model. The best of both models represents combining the data-driven and musculoskeletal models which offered best performance. The boxes extend from the lower to upper quartile values of the data, with a line at the median and a dot at the mean. The whiskers extend to the last data point within 2.5 times the interquartile range. Fliers were not plotted due to the wide range in errors.

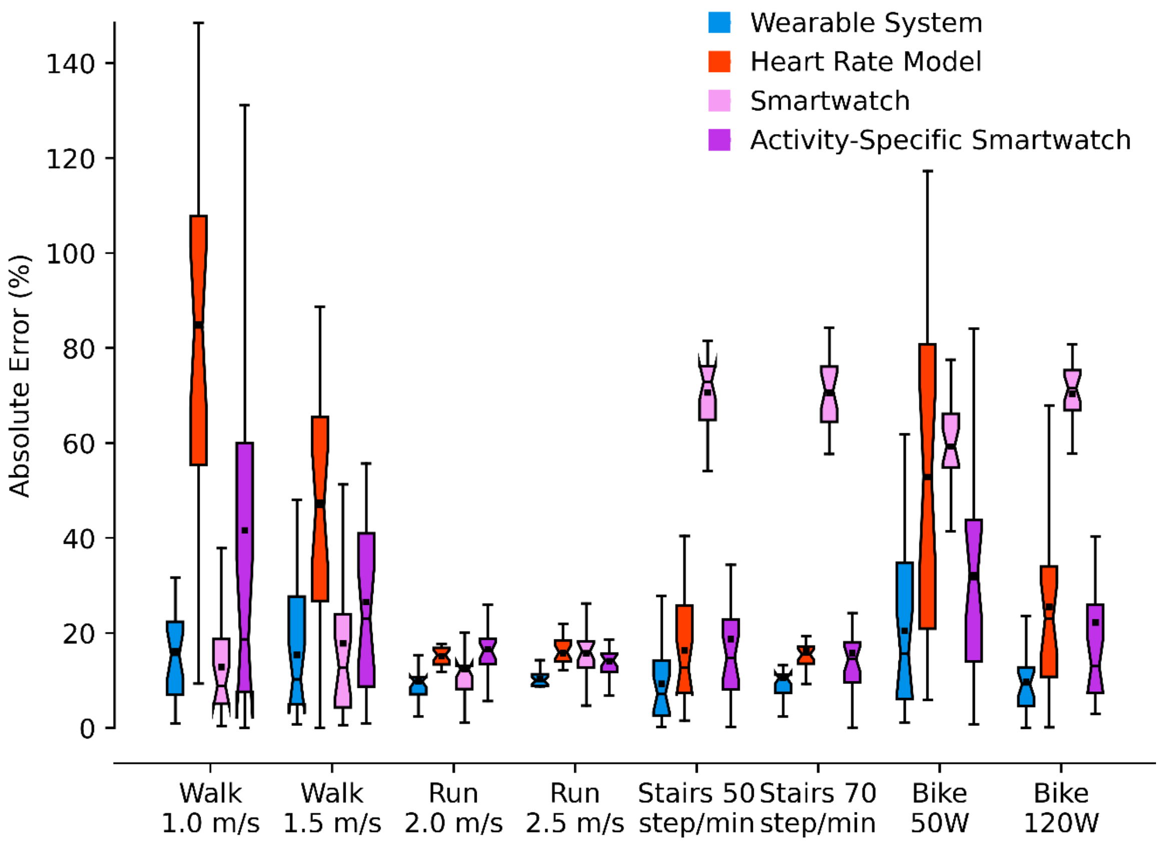

Supplementary Fig.7. Steady-state errors for each activity and method. The Wearable System had consistently low errors. The other methods accuracy varied by activity. The Heart Rate Model and Activity-Specific Smartwatch had the largest errors during walking and biking. The Smartwatch had drastically increased errors the stair climbing and biking activities. The number of subjects that completed each respective condition were: 24, 24, 13, 11, 22, 14, 24, and 14. The boxes extend from the lower to upper quartile values of the data, with a line at the median and a dot at the mean. The whiskers extend to the last data point within 2.5 times the interquartile range. Fliers were not plotted due to the wide range in errors.

Vertical Force (1)	Kinematics (3)	EMG (7)	IMU (4)	Relative Error (%)
X	X	X	X	**10.5**
X				19.1
	X			36.3
		X		46.8
			X	13.7
X	X			24.3
X		X		17.5
X			X	11.5
	X	X		28.6
		X	X	12.4
	X		X	12.4
X	X		X	10.6
X		X	X	**10.5**
X	X	X		20.2
	X	X	X	11.5

Supplementary Table 1. The Data-Driven Model results for permutations of input sensor classes. Sensor classes include: vertical ground reaction force (Vertical Force), lower-limb joint kinematics (Kinematics), electromyography (EMG), and inertial measurement units (IMU). The number next to the sensor classes indicates the number of sensors used for that class. The individual class with the lowest error was IMU and in general the IMUs paired with other sensors had lower error than the other sensors alone. The error was computed using the cross-validation approach over all permutations of subjects and conditions.

Location of IMU Placements				
**Hip**	**Thigh**	**Shank**	**Foot**	**Relative Error (%)**
X	X	X	X	**13.7**
X				16.8
	X			16.7
		X		17.1
			X	17.4
X	X			14.7
X		X		16.0
X			X	17.9
	X	X		**13.7**
		X	X	15.7
	X		X	14.9
X	X		X	14.6
X		X	X	14.5
X	X	X		14.8
	X	X	X	15.4

Supplementary Table 2. The Data-Driven Model results for permutations of inertial measurement units (IMUs) placed at different locations on one leg. The IMU placements included the hip, thigh, shank, and foot. The permutation with the lowest error was with one IMU worn on the thigh and shank. The error was computed using the cross-validation approach over all permutations of subjects and conditions.

Computation steps	Time (s)
1. Pre-processing data	0.005
2. Linear regression model estimation	0.004
The Wearable System total	0.009
1. Pre-processing data	0.004
2. OpenSense inverse kinematics	2.720
3. Linear regression model estimation	0.002
4. OpenSim forward dynamics simulation	76.290
The Musculoskeletal Model total	79.016

Supplementary Table 3. Computation time for the Wearable System and the Musculoskeletal Model. Each estimate was computed by processing an individual stride of data. The Wearable System estimate was computed with a single core of a Raspberry Pi 3b+ and the Musculoskeletal Model was computed using a single core of an i7 laptop processor. The time required to train the linear regression model and scale the Musculoskeletal Model occurred offline and was not included in the total. The Wearable System estimation required less than 0.01 seconds, feasible for real-time use.

Question text (1 = Strongly Agree, 2 = Somewhat Agree, 3 = Neither Agree Nor Disagree, 4 = Somewhat Disagree, 5 = Strongly Disagree)	Mean ± Std
I think that I would like to use this system frequently.	2.8 ± 1.1
I found the system unnecessarily complex.	4.4 ± 0.8
I thought the system was easy to use.	1.6 ± 0.6
I think that I would need the support of a technical person to be able to use this system.	4.5 ± 0.5
I found the various functions in this system were well integrated.	1.6 ± 0.8
I thought there was too much inconsistency in this system.	4.4 ± 0.8
I would imagine that most people would learn to use this system very quickly.	1.6 ± 0.8
I found the system very cumbersome to use.	3.8 ± 1.1
I felt very confident using the system.	1.8 ± 0.9
I needed to learn a lot of things before I could get going with this system.	4.6 ± 0.6
Total usability score (out of 100)	80.9 ± 7.2

Supplementary Table 4. System usability survey results. This questionnaire is the standard System Usability Scale (*40*). It is a Likert scale meant to evaluate the usability of a system. 21 participants evaluated the Wearable System with this survey. The Wearable System had a relatively high usability score of 80.9 averaged across participants.

Prompt: Overall reactions to the Wearable System	Mean ± Std
0 (heavy) to 9 (light)	7.3 ± 1.3
0 (obtrusive) to 9 (unobtrusive)	5.9 ± 2.3
0 (complex) to 9 (simple)	7.0 ± 1.9
0 (painful) to 9 (comfortable)	7.3 ± 1.1
0 (bulky) to 9 (compact)	6.6 ± 2.0

Supplementary Table 5. System comfort survey results. This questionnaire was based on the Questionnaire for User Interaction Satisfaction survey (*42*). 21 participants were asked to evaluate the Wearable System on a scale of 0 to 9 for different comfort related metrics. The Wearable System had high values, indicating it was comfortable to wear and use.
