# Supplementary material for: Sensing leg movement enhances wearable monitoring of energy expenditure
Source: Nat Commun. 2021 Jul 13;12:4312. doi: 10.1038/s41467-021-24173-x (PMC8277831; doi:10.1038/s41467-021-24173-x)
Supplement: Supplementary file 3 — Description of Additional Supplementary Files [file 41467_2021_24173_MOESM3_ESM.docx]

**Description of Additional Supplementary Files**

File Name: Supplementary Movie 1.

Description: The Wearable System estimating energy expenditure during free-living. The Wearable System components are shown and described. The Wearable System estimates energy expenditure in an outdoor setting during a rapid succession of activities including stair climbing, walking, running, and biking. The real-time estimates are visualized as the person is shown to take each step.

File Name: Supplementary Movie 2.

Description: Energy expenditure estimation during steady-state conditions. The experimental setup for all steady-state conditions are shown. The energy expenditure estimates are shown for all methods from the start of one steady-state condition. This visualizes the rate at which the estimates converge to steady-state.

File Name: Supplementary Movie 3.

Description: Energy expenditure estimation during time-varying conditions. The experimental setup for two of the time-varying conditions are shown: transitioning between quiet standing and walking as well as sinusoidally-varying between walking and running. The real-time estimates are visualized as the person is shown to take each step compared to the ground truth Interpolated Respirometry.

File Name: Supplementary Movie 4.

Description: Donning and doffing the Wearable System. This video demonstrates the Wearable System being donned in 25 seconds and doffed in 14 seconds. The simple procedure helps inform intuition into how it may be used in clinics or at-home.
