# Supplementary material for: Sensing leg movement enhances wearable monitoring of energy expenditure
Source: Nat Commun. 2021 Jul 13;12:4312. doi: 10.1038/s41467-021-24173-x (PMC8277831; doi:10.1038/s41467-021-24173-x)
Supplement: Supplementary file 8 — Reporting Summary [file 41467_2021_24173_MOESM8_ESM.pdf]

nature research

Corresponding author(s): Patrick Slade

Last updated by author(s): May 19, 2021

Reporting Summary

Nature Research wishes to improve the reproducibility of the work that we publish. This form provides structure for consistency and transparency in reporting. For further information on Nature Research policies, see our Editorial Policies and the Editorial Policy Checklist.

Statistics

For all statistical analyses, confirm that the following items are present in the figure legend, table legend, main text, or Methods section.

n/a	Confirmed	
☐	☒	The exact sample size (n) for each experimental group/condition, given as a discrete number and unit of measurement
☐	☒	A statement on whether measurements were taken from distinct samples or whether the same sample was measured repeatedly
☐	☒	The statistical test(s) used AND whether they are one- or two-sided<br>*Only common tests should be described solely by name; describe more complex techniques in the Methods section.*
☒	☐	A description of all covariates tested
☐	☒	A description of any assumptions or corrections, such as tests of normality and adjustment for multiple comparisons
☐	☒	A full description of the statistical parameters including central tendency (e.g. means) or other basic estimates (e.g. regression coefficient) AND variation (e.g. standard deviation) or associated estimates of uncertainty (e.g. confidence intervals)
☐	☒	For null hypothesis testing, the test statistic (e.g. F, t, r) with confidence intervals, effect sizes, degrees of freedom and P value noted<br>*Give P values as exact values whenever suitable.*
☒	☐	For Bayesian analysis, information on the choice of priors and Markov chain Monte Carlo settings
☒	☐	For hierarchical and complex designs, identification of the appropriate level for tests and full reporting of outcomes
☒	☐	Estimates of effect sizes (e.g. Cohen's d, Pearson's r), indicating how they were calculated

Our web collection on statistics for biologists contains articles on many of the points above.

Software and code

Policy information about availability of computer code

Data collection	Data analysis was performed using Python version 3.6.1, Matlab 2019a, Motion Analysis 7.1, and the Apple Health app 4.3.2. The required python packages are numpy (1.17.4), scikit-learn (0.21.3), scipy (1.3.2), matplotlib (2.0.2), natsort (6.2.0), jupyter (1.0.0), ipython (5.3.0), and pands (0.25.3). Software scripts used for data collection are available in a public repository: https://simtk.org/projects/energy-est
Data analysis	Data analysis was performed using Python version 3.6.1. The required python packages are numpy (1.17.4), scikit-learn (0.21.3), scipy (1.3.2), matplotlib (2.0.2), natsort (6.2.0), jupyter (1.0.0), ipython (5.3.0), and pands (0.25.3). Software scripts used for data analysis are available in a public repository: https://simtk.org/projects/energy-est

For manuscripts utilizing custom algorithms or software that are central to the research but not yet described in published literature, software must be made available to editors and reviewers. We strongly encourage code deposition in a community repository (e.g. GitHub). See the Nature Research guidelines for submitting code & software for further information.

Data

Policy information about availability of data

All manuscripts must include a data availability statement. This statement should provide the following information, where applicable:

- Accession codes, unique identifiers, or web links for publicly available datasets
- A list of figures that have associated raw data
- A description of any restrictions on data availability

All data necessary to replicate this work are available in a public repository: https://simtk.org/projects/energy-est. This includes experimental data including the different models, data to train the Data-Driven Model used by the wearable system, data to validate the wearable system results, and data to replicate the wearable system.

Field-specific reporting

Please select the one below that is the best fit for your research. If you are not sure, read the appropriate sections before making your selection.

- [x] Life sciences
- [ ] Behavioural & social sciences
- [ ] Ecological, evolutionary & environmental sciences

For a reference copy of the document with all sections, see nature.com/documents/nr-reporting-summary-flat.pdf

Life sciences study design

All studies must disclose on these points even when the disclosure is negative.

Sample size	The first study design for the first three experiments used to create the Wearable System are discussed in the Methods. The necessary sample size to validate the Wearable System was found to be 15 subjects from a power analysis based earlier experiments which estimated the Wearable System would have 14% absolute error, a standard deviation of 12%, a difference of at least 14% with each compared method, and a power of 0.9. We stopped data collection after reaching 25 subjects in case of sensor failures and because some subjects were not able to complete all conditions.
Data exclusions	One subject was excluded because of sensor failure and no other exclusions were made.
Replication	We have made the experimental data available and provided all code used to replicate estimates from the Wearable System and any other models used in the study.
Randomization	All participants completed the same set of experimental conditions which were randomized.
Blinding	All subjects were in the same group during the experiment, so the blinding was not relevant to the study.

Reporting for specific materials, systems and methods

We require information from authors about some types of materials, experimental systems and methods used in many studies. Here, indicate whether each material, system or method listed is relevant to your study. If you are not sure if a list item applies to your research, read the appropriate section before selecting a response.

Materials & experimental systems

n/a	Involved in the study	
x		Antibodies
x		Eukaryotic cell lines
x		Palaeontology and archaeology
x		Animals and other organisms
	x	Human research participants
x		Clinical data
x		Dual use research of concern

Methods

n/a	Involved in the study	
x		ChIP-seq
x		Flow cytometry
x		MRI-based neuroimaging

Human research participants

Policy information about studies involving human research participants

Population characteristics	There were four separate experiments completed in this study. The first three tests used to develop the Wearable system are listed in the methods. The experiment that validated the accuracy of the Wearable System was from a population of adults with characteristics: n = 24, 15 men and 9 women; age = 34.8 ± 11.6 yr; body mass = 74.3 ± 13.1 kg; height = 1.73 ± 0.07 m; body mass index = 24.9 ± 4.1.
Recruitment	The participants were recruited in order to represent the adult population in the U.S. in terms of the mean and standard deviation in physiological features such as height, weight, age, and body mass index distributions. The participants had a smaller age and weight range than the general population, thus evaluating the methods with a more physically capable group of participants than the general adult demographics. Participants collected after March 2020 were required to be Stanford affiliates due to the added health risks of human subject testing during the Covid-19 pandemic.
Ethics oversight	Stanford University Institutional Review Board (IRB-17282)

Note that full information on the approval of the study protocol must also be provided in the manuscript.
